# Susceptibility pattern of *Mycobacterium tuberculosis* over a period of five years at Indus Hospital and Health Network, Karachi, Pakistan

**DOI:** 10.12669/pjms.38.ICON-2022.5778

**Published:** 2022-01

**Authors:** Nazia Khursheed, Sunil Asif, Safia Bano, Maria Mushtaq Ali, Fareeha Adnan

**Affiliations:** 1 Dr. Nazia Khursheed, FCPS Microbiology. Department of Microbiology, Indus Hospital and Health Network, Korangi Campus, Karachi, Pakistan; 2 Sunil Asif, F.Sc. Department of Microbiology, Indus Hospital and Health Network, Korangi Campus, Karachi, Pakistan; 3 Safia Bano, M.Phil. Department of Microbiology, Indus Hospital and Health Network, Korangi Campus, Karachi, Pakistan; 4 Maria Mushtaq Ali, M.Sc in Biochemistry. Indus Hospital Research Center, Indus Hospital and Health Network, Korangi Campus, Karachi, Pakistan; 5 Fareeha Adnan, FCPS Microbiology. Department of Microbiology, Indus Hospital and Health Network, Korangi Campus, Karachi, Pakistan

**Keywords:** *Mycobacterium tuberculosis*, Drug-resistance, Pulmonary and Extrapulmonary tuberculosis

## Abstract

**Objective:**

To determine the susceptibility pattern and frequency of isolation of multidrug, pre-extensively drug and extensively drug resistant TB in a tertiary care hospital in Karachi, Pakistan.

**Method:**

A cross-sectional study was designed. Samples received in the lab were processed for growth and sensitivity testing of Mycobacterium tuberculosis. Isolation of MTB was done on Mycobacteria growth indicator tube (MGIT) followed by identification using MPT64. Samples were than evaluated for drug sensitivity against first and second-line antimycobacterial drugs. Statistical analysis was performed using SPSS version 24.0.

**Results:**

Of the 20014 samples received, 23.1% were identified as Mycobacterium tuberculosis. Drug sensitivity testing was performed on 95.9% isolates. Fifty-two percent samples were from males and 48% female patients. The study found statistically non-significant relationship between gender and likelihood of disease with drug-resistant (DR)-MTB organisms. The rate of isolation of MDR-TB was highest (43%) among ages 25-55 years and previously treated patients compared to newly diagnosed patients (62% vs 36%). Among MTB positive samples, 91.5% were pulmonary while 8.5% were extrapulmonary samples. Extrapulmonary samples were more likely to be sensitive to antimycobacterial drugs. The highest resistance was observed against Isoniazid (pulmonary=58%; extrapulmonary=12.7%), Rifampicin (pulmonary=58.7%; extrapulmonary=8.2%), and Levofloxacin (pulmonary=29.2%; extrapulmonary=20%).

**Conclusion:**

A considerable number of drug resistant tuberculosis cases were identified in the present study. It is essential to develop further strategies to reduce the spread of this disease.

## INTRODUCTION

Mycobacterium tuberculosis (MTB) is the 9th leading cause of death with annually 10 million new cases and 1.7 million deaths worldwide.^1^ Among 30 high TB burden countries Pakistan has been ranked at fifth position according to WHO Report 2020.^2^ According to WHO Eastern Mediterranean Regional Office (EMRO) reports (2019), there were approximately 265 incidences of TB per 100,000 population in Pakistan. Moreover, there were 15, 5537 (new and previously treated) cases of TB reported during 9 months in 2020 in Pakistan. Although, TB is a curable and preventable disease, with 85% cure rate,^3^ emergence of Multidrug resistance (MDR) strains worsens the situation. Pakistan ranks fourth among the 27 high MDR-TB burden countries.^4^

In Pakistan, the per capita cost for the treatment of TB is $307.74. In this scenario, surveillance study will be helpful in evaluating the effectiveness of TB prevention and control programs and highlight the emerging resistance pattern in the community. Surveillance of frequency and drug resistance profile provide clinicians with a background of MTB drug susceptibility and assist in designing modifications in their approach to DR-TB transmission control and in creating effective regimens and treatment strategies for DR-TB patient management based on WHO guidance. Taking in to account the above context, the present study is designed to analyze the frequency and susceptibility pattern of MTB in clinical isolates from 2016 to 2020 at the Indus Hospital and Health Network (IHHN), Korangi campus, Karachi, Pakistan.

## METHODS

This retrospective study was conducted at the IHHN, located in Karachi, Pakistan. It has supported tuberculosis control efforts in Pakistan since 2007. The study was approved by the Institutional Review Board (IRB) of IHHN (study number IHHN_IRB_2021_04_019).

All MTB isolates from pulmonary and extra-pulmonary samples received during 2016−2020 were processed for culture and drug susceptibility testing (DST). Data were extracted using the Hospital Management Information System (HMIS), which included patient medical record number, age, gender, date of isolation, specimen-type and susceptibility pattern of TB against first- and second-line mycobacterial agents.

### Microbiological methods:

All specimens were processed at the Microbiology Laboratory of IHHN. The specimens were digested and decontaminated using the N-acetyl-L-cysteine-NaOH method and smears were prepared according to the standard protocols, subjected to auramine O staining and examined under Light Emitting Diode (LED) fluorescence microscope. For the growth of *M. tuberculosis*, specimens were inoculated on both Mycobacteria growth indicator tube (MGIT) and Lowenstein Jensen (LJ) medium (Becton Dickinson). Growth from the positive LJ slant and MGIT vials were stained with Ziehl nelson. Identification of *M. tuberculosis* was done by MPT64.

### Drug Susceptibility Testing:

Susceptibility testing was performed using BACTEC MGIT 960 method by following the standard procedure of the manufacturer.^5^ Final drug concentrations used were 1.0 µg/ml for streptomycin, 0.1 µg/ml for isoniazid, 1.0 µg/ml for rifampicin, 5.0 µg/ml for ethambutol and for pyrazinamide 100 µg/ml, for kanamycin and capreomycin 2.5 µg/ml, amikacin 1 µg/ml, ofloxacin 2 µg/ml, moxifloxacin 1 µg/ml, 0.25 µg/ml and levofloxacin at 1 µg/ml. The relative growth ratio was determined by comparing the fluorescence between the two tubes by the system’s software algorithm. Susceptibility results were determined by comparison of analysis of fluorescence in the drug-containing tubes and Growth Control tube. To interpret the results for first line and second line drugs, the standard protocol recommended by manufacturers was followed for DST by the MGIT 960 method. When the growth unit (GU) of the growth control reaches 400 within 4–13 days, the GU values of the drug-containing vials were assessed.; the result was reported as susceptible and resistant strains, when the GU of the drug-containing tubes were found to be 100 and >100 respectively.

### Quality controls:

Quality control of each batch of culture and drug susceptibility pattern (DST) performed with the reference strain H37Rv (ATCC 27294), which is susceptible for all standard anti-tuberculosis drugs.

### Statistical analysis:

The data were analyzed by using SPSS 24.0.^6^ Quantitative variables were calculated as mean ± std, whereas, descriptive statistics were used to calculate the frequency and percentage of drug resistance tuberculosis cases by age, gender and history of patients. Comparisons of categorical variables were performed by using χ2 test. P-value <0.05 was considered as statistically significant.

## RESULTS

Twenty thousand and fourteen samples were received during the study period. Among them 4652 (23.1%) were flagged positive as Mycobacterium species, 14836 (74.1%) were negative, 519 (2.5%) were contaminated and 7 (0.03%) were rejected according to the rejection criteria of the laboratory. Out of 4652 positive samples, DST was performed on 4463 (95.9%) isolates identified as MTB, 115 (2.4%) were identified as MOTT (Mycobacterium other than MTB), while 74 (1.5%) were MTB positive with contamination. These 189 samples were excluded from the analysis. The frequency of MDR, pre-extensively drug resistant (pre-XDR), extensively drug resistant (XDR) and sensitive Mycobacterium tuberculosis were found to be 40%, 15%, 1% and 44% respectively.

Among positive samples male preponderance was found in 2327 (52.1%) samples and 2136 (47.9%) samples were from females. Mean age of the sampled patients was 33 (std ± 16.7) years with minimum <1 year and maximum 95 years. The pulmonary samples were 4086 (91.5%) and extra pulmonary samples were 377 (8.5%). Of the positive samples those from newly enrolled patients were 3743 (83.8%) and previously treated patients were 706 (15.8%).

Among MDR-TB cases, comparison has been done between age groups < 15, 15-24, 25-34, 35-44, 45–55 and above 55 years. Age groups 25-34, 35-44 and 45-55years were the most affected groups with MDR-TB as compared to other age groups (Table-I). MDR-TB, pre-XDR and XDR isolates were statistically more prevalent in previously treated than in newly diagnosed patients, shown in (Table-I).

**Table I:** Association of types of TB and Demographics.

	MDR (%)	Pre-XDR (%)	XDR (%)	Non-MDR (%)	Total	P value
**Type of TB**						
PTB	1767 (43.2)	650 (15.9)	28 (0.68)	1641 (40.1)	4086	0.000
EPTB	25 (6.6)	8 (2.1)	0 ()	344 (91.2)	377	
Total	1792	658	28	1985	4463	
**Gender**						
Male	948 (40.7)	33 (14.3)	17 (0.73)	1029 (44.2)	2136 (100)	0.586
Female	844 (39.5)	325 (15.2)	11 (0.51)	956 (44.7)	2327 (100)	
**History of treatment**						
Previously Treated	439 (62.1)	152 (21.5)	11 (1.5)	104 (14.7)	706 (100)	0.000
Newly Enrolled	1348 (36)	499 (13.3)	17 (0.45)	1879 (50.2)	3743 (100)	
Unknown	5	7	0	2	14	
**Age Groups**						
<15	147 (37.5)	38 (9.7)	0 (0)	206 (52.6)	391 (100)	0.000
15-24	475 (37.8)	185 (14.7)	6 (0.47)	588 (46.8)	1254 (100)	
25-34	416 (43.0)	154 (15.9)	6 (0.62)	390 (40.3)	966 (100)	
35-44	269 (43.3)	92 (14.8)	6 (0.96)	254 (40.9)	621 (100)	
45-55	290 (43.6)	106 (15.9)	4 (0.60)	264 (39.7)	664 (100)	
>55	195 (34.3)	83 (14.6)	6 (1.0)	283 (49.9)	567 (100)	

Among PTB samples, tested for first-line drugs, showed highest resistance against Rifampicin (58.7%) and Isoniazid (58.0%) followed by Pyranzinamide (20.7%), Ethambutol (13.0%), and Streptomycin (12.4%). For second-line drugs, high resistance was observed for Ofloxacin (30.2%) and Levofloxacin (29.2%) (Fig.1a).

**Fig.1a:**
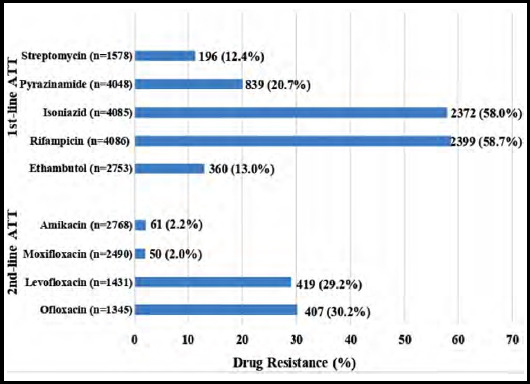
Antibiotic susceptibility pattern of MTB in PTB samples.

Extra pulmonary samples showed high sensitivity as compared to pulmonary samples. In EPTB samples highest resistance was observed for Isoniazid (12.7%), followed by Rifampicin (8.2%), Streptomycin (5.0%), and Pyrazinamide (4.5%) among first line antituberculosis drugs. In second-line drug similar to PTB samples, highest resistance was observed against Levofloxacin (20%). However, EPTB samples showed 100% sensitivity to Ethambutol and Moxifloxacin (Fig.1b).

**Fig.1b:**
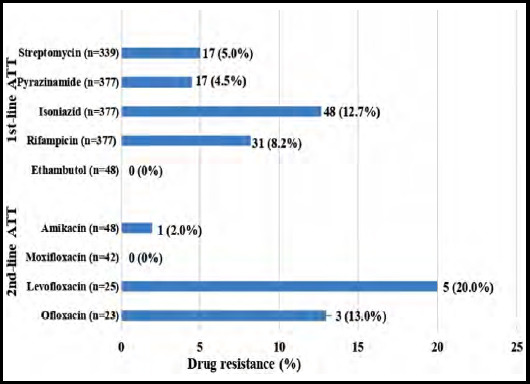
Antibiotic susceptibility pattern of MTB in EPTB samples.

## DISCUSSION

This study reports the susceptibility pattern of MTB after laboratory record review. The frequency of isolation of MTB was found to be 23.2% (4652/20014) in the current study. Whereas a study conducted in Punjab reported low incidence rate of 14%, on the contrary, 0.002 incidence rate was observed from developed countries.^7^

Our study reports a high rate of MDR–TB isolates (40%) which is analogous with a local study which reported a rate of 38.7%.^8^ Inconsistent with our findings, a study done in Punjab reported only 9.3% of MDR-TB. The high prevalence of TB in Karachi (Sindh) as compared to Punjab might be because Karachi is a densely populated city and also an economic hub where people come from many different regions, often live in close quarters, and this increases TB transmission in the community. The Indus Hospital has been an MDR-TB referral and treatment site since 2007 another reason for a higher rate of MDR-TB. Multiple studies from different regions of the world have reported 4-50% incidence rate of MDR-TB.^9^

Among first line antimycobacterials, the highest resistance was observed for Rifampicin and Isoniazid. The findings in present study are similar to the studies conducted in Bangladesh and South Africa that showed high rates of resistance to Isoniazid and Rifampicin.^10^,^11^ However the high rates of resistance to streptomycin and Isoniazid observed in these studies is not in concordance with our results.^11^ An alarming finding is the high resistance observed against Levofloxacin (29.2%) and Ofloxacin (30.2%), as quinolones are important components of DR-TB regimens. The reason for this upsurge is probably excessive and unregulated use of these antibiotics.

Ineffectively managed MDR TB cases mutate into XDR-TB. Our data showed 15% pre-XDR-TB and 1% XDR-TB strains. Our results are similar to studies conducted in India for instance Sharma and colleagues reported 18.4% pre-XDR prevalence.^12^ In comparison, a study conducted by Sameer Adwani et al., from India, from a similar population density and setting as Karachi, reported a higher prevalence of pre-XDR-TB (55.9%) and XDR-TB (4.8%).^13^ The emergence of pre-XDR and XDR strains is alarming since treatment options are limited, costly and outcomes are poor.

In this study no statistical significance was found between gender and different categories of drug resistance. This is consistent with a study done by Aarain et al.^10^ In contrast, a study conducted in Bangladesh reported three times higher TB prevalence in males.^14^ A study in Israel also stated males as a risk factor to DR-TB.^15^ Another study conducted in Karachi reported high drug resistance in male gender which is not in accordance with our findings. The significance of gender with respect to drug resistance needs to be further explored. The conflicting results was observed with higher degree of vulnerability of the female gender to DR-TB in Pakistan and the Republic of Georgia.^16^,^17^

In contrast to gender, age was found to be associated with drug resistant TB. The highest frequency of MDR- TB was observed in the age group of 24 -55 years. Previous studies documented a higher rate of MDR- TB in the 10-25 year age group.^18^ Our results can be supported by the fact that this age group is more exposed to the community consequently, making them more vulnerable. It might also be accredited to other activities connected to the phase of development where social and cultural factors gain importance in their lives, financial constraints, and TB-related stigma. In addition, a lower resistance in under five and above 55 years of age is possibly due to lower level of exposure and inactive lifestyle which subsidizes the risk of cross-transmission of MDR strains. In contrast to our findings, some investigations have not shown any association between the risk of MDR-TB and age.^19^-^21^

In the current study, the overall occurrence of PTB was 91.5%, similar to the results observed in Chiniot, where 90.5% of cases were reported as PTB, significantly higher percentage compared to other studies (40% to 44% cases) conducted in Khyber Pakhtunkhwa, Pakistan.^22^ Furthermore, a study conducted in Ethiopia reported a 40.9% prevalence of PTB.^23^ According to the Global TB report EPTB contributes to 15% of the all TB cases.^24^ However, the proportion of EPTB among TB patients varies from country to country and ranges from 5% (China) to 29% (Afghanistan). Interestingly it also differs from province to province in Pakistan. The ratio is observed to be higher in Khyber Pakhtunkhwa and FATA, in comparison to Punjab and Sindh.^25^ Herein, of the total isolates 8.5% were EPTB. This proportion is lower than the developing countries such as US (21%), Italy (32%), and Australia (39%). These differences might be attributed to different factors including differences in socio-demographic features, failure of early diagnosis or under-estimation of EPTB in developing countries.^26^ Extrapulmonary-TB samples showed higher sensitivity (91.2%) to the antituberculosis drugs. This finding is further strengthened by another study conducted in Europe where a high rate of MDR-TB was associated with PTB. In India, a study conducted by Mourya et al reported a drug resistance rate of 13.4% in EPTB samples that is higher than the rate observed in the present study. On the contrary, a significantly low rate (2.2%) of MDR-TB in EPTB samples was reported in another study conducted in Pakistan.^25^ Higher sensitivity in EPTB samples might be because it does not contribute considerably to the transmission of the disease, consequently, there is a low chance of spreading drug resistance strains.

Collectively, the higher occurrence of MDR, pre XDR, and XDR TB was observed in previously treated patients. A similar pattern was observed in a meta-analysis done in Ethiopia for sub-Saharan African countries that reported the high prevalence of drug resistance TB strains in previously treated patients as compared to newly diagnosed cases.^27^ The major reason contributing to the high rate of drug resistance in previously treated patients is non- adherence to antituberculosis treatment strategies. While secondary reasons could be low literacy levels, discriminatory behavior by health care providers, deferrals in care seeking behavior and self-denial due to disgrace experienced by TB patients.

Although, different parts of the world reported variable patterns of drug-resistant TB, these dynamics in the resistance profile may be due to very different modes of living habits, poor application of TB infection control policy, inadequate and irregular treatment, and the dissemination of drug-resistant strains.

### Strength:

The strength of this study is that it is sharing the current frequency and susceptibility pattern of *MTB*. This information can be used to observe evolving patterns of drugs resistance which could help in modifying treatment guidelines.

### Limitation:

The main limitation of this study is the retrospective collection of data. For the majority of patients, we were not able to retrieve a detailed clinical history, X-ray findings, and treatment outcome. Another limitation of our study is that it is a single centre study, therefore, it cannot be generalized for the whole population and possesses selection bias.

## CONCLUSION

The ongoing transmission of DR-TB strains in the community is a serious public health problem as treatment is often prolonged and newer drugs are not easily available and costly making treatment completion difficult to achieve. The major risk factors for the development of DR-TB identified in the present study are age and history of previous TB treatment. Hence, there is an urgent need for surveillance programs, availability of new molecular tests at district level, effective TB programs, extensive research to explain the factors concomitant with DR-TB.

### Authors’ Contribution:

**NK** Conceived, designed and critically review of manuscript.

**SA and SB** Did data collection and analysis.

**MMA** Did data analysis and manuscript writing.

**FA** Did critical review of manuscript.

**NK** Takes the responsibility and accountable for all aspects of the work in ensuring that questions related to the accuracy or integrity of any part of the work are appropriately investigated and resolved.
